# Imported Chikungunya Virus Strains, Taiwan, 2006–2009

**DOI:** 10.3201/eid1511.090398

**Published:** 2009-11

**Authors:** Jyh-Hsiung Huang, Cheng-Fen Yang, Chien-Ling Su, Shu-Fen Chang, Chia-Hsin Cheng, Sheng-Kai Yu, Chien-Chou Lin, Pei-Yun Shu

**Affiliations:** Centers for Disease Control, Taipei, Taiwan, Republic of China

**Keywords:** Chikungunya, chikungunya virus, Taiwan, E1-226V, phylogenetic analysis, viruses, letter

**To the Editor:** Chikungunya is a reemerging infectious disease that is endemic to Africa and Asia and caused by a mosquito-borne alphavirus in the family *Togaviridae*. Previous phylogenetic studies showed that chikungunya virus (CHIKV) strains were clustered into 3 distinct genotypes separated primarily by location into West African, Central/East/South African, and Asian genotypes (*1*,*2*).

Earlier outbreaks in Thailand, Cambodia, Vietnam, Myanmar, the Philippines, Malaysia, Indonesia, Pakistan, and India during 1960–1999 were caused by strains of the Asian genotype (*2*). However, explosive epidemics in Indian Ocean islands and India since 2005 and the worldwide increase in travel have changed the distribution of CHIKV genotypes. Recent studies have shown that different lineages of CHIKV strains of the Central/East/South African genotype have expanded locally and spread to new areas in Africa, Europe, and Asia and caused epidemics (*2*–*7*).

Imported chikungunya cases were identified at airports by active surveillance (fever screening) in Taiwan (*3*). Among 14,289 febrile patients arriving at Taiwan Taoyuan International Airport from January 2006 through February 2009, a total of 13 were confirmed to have CHIKV infections. One additional chikungunya case was detected at Kaohsiung International Airport among 801 febrile patients from February 2008 through February 2009. These imported cases were introduced from Indonesia (7 cases), Malaysia (4 cases), Singapore (1 case), Bangladesh (1 case), and India (1 case). Real-time quantitative reverse transcription–PCR showed virus titers ranged from 10^3.6^ PFU/mL to 10^6.4^ PFU/mL for day 1–3 acute-phase serum samples from these patients. CHIKV strains were successfully isolated by using a cell culture (C6/36) method (Technical Appendix).

To identify genetic relationships among these 14 imported CHIKV isolates, complete structural polyprotein gene sequences of 10 isolates (GenBank accession nos. FJ807886–FJ807895) and full genome sequences of 4 isolates (Singapore/0611aTw, Indonesia/0706aTw, Bangladesh/0810aTw, and Malaysia/0810bTw strains) (GenBank accession nos. FJ807896–FJ807899) were determined. Nucleotide sequences of complete open reading frames of Singapore/0611aTw, Bangladesh/0810aTw, and Malaysia/0810bTw isolates were most closely related to the India IND-06-AP3 strain (99.95%, 99.84%, and 99.77% identities, respectively) and other India 2006 isolates, which suggests common genetic origins from India.

In comparison with other CHIKV strains, unique substitution K252Q in the envelope 2 (E2) protein was found in all 4 imported isolates from Malaysia, and 2 unique substitutions, V4A and N349D, in the envelope 1 (E1) protein were found in the imported Bangladesh/0810aTw isolate. The Indonesia/0706aTw isolate was most closely related to the Malaysia MY003IMR isolate (99.42% identity). A novel 4-aa deletion, corresponding to nonstructural protein 3 codons 379–382 (TTACCAACCATA coding for Leu-Pro-Thr-Ile in the Malaysia MY003IMR strain), was observed in the Indonesia/0706aTw strain when it was compared with other CHIKV sequences available in GenBank. Further sequence analysis showed that all 6 isolates from Indonesia had the same deletion in this region.

A phylogenetic tree based on 49 CHIKV partial E1 gene sequences was constructed to trace the origins of the 14 CHIKV strains reported in this study (Figure). Phylogenetic analysis shows that all 7 strains from Indonesia isolated during 2007–2008 are grouped into the Asian genotype and clustered in a distinct lineage. This lineage shows close relationship to the Malaysia/MY002IMR/2006 isolate. However, the 4 strains from Malaysia isolated during 2008–2009 belong to the Central/East/South African genotype and are clustered with CHIKV strains of India/ALP-6/2007, Italy/ITA07-RA1/2007, and Bangladesh/0810aTw/2008. These viruses also have the E1-A226V mutation. The imported Singapore/0611aTw/2006 and India/0812cTw/2008 strains belong to the Central/East/South African genotype and are clustered with several India/2006 (IND-06-AP3, IND-06-TN1 and DRDE-06), India/Kokkarayapalli/2008, and Singapore/EHIss622/2008 strains, which have an alanine at the position E1–226.

**Figure:**
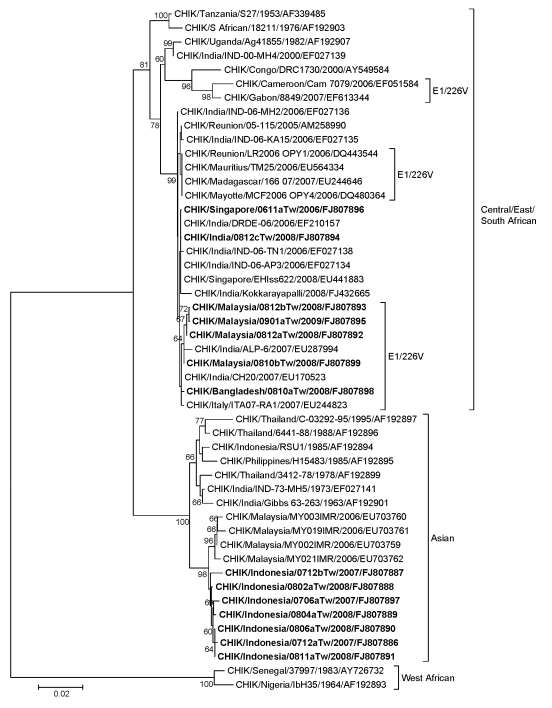
Phylogenetic relationships of chikungunya virus (CHIKV) isolates from 14 imported cases of chikungunya, Taiwan, 2006–2009. The tree was constructed on the basis of partial envelope 1 (E1) nucleotide sequences (836 bp, nt positions 10264–11099 of the prototype CHIKV S27 genomic sequence) of 49 CHIKV strains. Sequences obtained in this study are indicated in **boldface**. CHIKV strains with the E1-A226V mutation are indicated. Genotypes are indicated on the right. Viruses were identified by using the nomenclature of virus/country/strain/year of isolation/GenBank accession number. Analysis was performed by using MEGA 4 software and neighbor-joining (maximum composite likelihood) methods. Bootstrap support values >60 are shown (1,000 replicates). West African genotype Senegal strain 37997 sequence was used as the outgroup virus. Scale bar indicates nucleotide substitutions per site.

Our results provide insights into the current distribution of different CHIKV genotypes and lineages. Phylogenetic analysis demonstrated that CHIKV strains isolated from Indonesia during 2007–2008 remain stable and belong to the Asian genotype, whereas the other 7 isolates from Singapore, Bangladesh, Malaysia, and India belong to the Central/East/South African genotype. The Malaysia/2008–2009 and Bangladesh/2008 isolates have the E1–226(V) mutation similar to reported variants isolated in Cameroon, some Indian Ocean islands, India, Italy, and Gabon during 2006–2007 (*4*,*6*–*9*). These results show that different lineages of CHIKV strains from India with the Central/East/South African genotypes have been transmitted long distances by infected persons to various countries in Asia, including Singapore, Malaysia, and Bangladesh.

Although the urban mosquito *Aedes aegypti* is the primary vector for dengue and chikungunya transmission in Asia, the *Ae*. *albopictus* mosquito, a less efficient vector, was recently identified as the main or alternate vector in chikungunya outbreaks in central and East Africa (*4*,*7*,*10*), India (*8*), and Italy (*6*). Recent studies have suggested that the increased chikungunya outbreaks caused by CHIKV strains of the Central/East/South African genotype might be associated with a change in 1 nt, the A226V mutation, in the E1 protein during continuous epidemics (*8*,*9*). It is not known whether E1-A226V variants play a dominate role in urban or periurban areas of Asia and Africa where *Ae*. *aegypti* and *Ae*. *albopictus* mosquitoes are present.

## Supplementary Material

Technical Appendix Data and diagnostic test results for 14 imported chikungunya cases, Taiwan, 2006-2009*
