# Supplementary material for: Imported Chikungunya Virus Strains, Taiwan, 2006–2009
Source: Emerg Infect Dis. 2009 Nov;15(11):1854–6. doi: 10.3201/eid1511.090398 (PMC2857229; doi:10.3201/eid1511.090398)
Supplement: Technical Appendix — Data and diagnostic test results for 14 imported chikungunya cases, Taiwan, 2006-2009* [file 09-0398_Techapp-s1.pdf]

Publisher: CDC; Journal: Emerging Infectious Diseases
Article Type: Letter; Volume: 15; Issue: 11; Year: 2009; Article ID: 09-0398
DOI: 10.3201/eid1511.090398; TOC Head: Letter

Imported Chikungunya Virus Strains, Taiwan, 2006–2009

Technical Appendix

Table. Data and diagnostic test results for 14 imported chikungunya cases, Taiwan, 2006–2009*

Case no.	Date†	Country‡	Days postonset of illness§	Real-time qRT-PCR result, PFU/mL	IgM/IgG ELISA¶	IFA titer (IgM + IgG + IgA)	Virus strain	Virus genotype	E1–226	GenBank accession no.
1	2006 Nov 20	Singapore	2, 17	$10^{5.9}$, ND	0.257/0.090, 3.164/2.010	<10, 640	0611aTw	C/E/S African	A	FJ807896
2	2007 Jun 21	Indonesia	3, 20	$10^{4.7}$, ND	0.615/0.084, 3.184/1.845	<10, 640	0706aTw	Asian	A	FJ807897
3	2007 Dec 28	Indonesia	2, 9	$10^{5.7}$, ND	0.072/0.085, 1.056/0.063	<10, 80	0712aTw	Asian	A	FJ807886
4	2007 Dec 30	Indonesia	1, 17	$10^{5.7}$, ND	0.130/0.067, 2.450/0.353	<10, 320	0712bTw	Asian	A	FJ807887
5	2008 Feb 12	Indonesia	2, 31	$10^{6.0}$, ND	0.178/0.070, 1.760/0.306	<10, 640	0802aTw	Asian	A	FJ807888
6	2008 Apr 19	Indonesia	1, 6	$10^{4.7}$, ND	0.078/0.072, 0.760/0.082	<10, 80	0804aTw	Asian	A	FJ807889
7	2008 Jul 5	Indonesia	3	$10^{5.2}$	0.114/0.073	<10	0806aTw	Asian	A	FJ807890
8	2008 Oct 16	Bangladesh	3	$10^{6.4}$	0.103/0.072	<10	0810aTw	C/E/S African	V	FJ807898
9	2008 Oct 22	Malaysia	1, 14	$10^{5.4}$,ND	0.060/0.068, 2.207/0.353	<10, 320	0810bTw	C/E/S African	V	FJ807899
10	2008 Nov 26	Indonesia	2, 14	$10^{3.6}$, ND	0.120/0.073, 2.800/0.545	<10, 640	0811aTw	Asian	A	FJ807891
11	2008 Dec 8	Malaysia	1	$10^{5.2}$	0.106/0.063	<10	0812aTw	C/E/S African	V	FJ807892
12	2008 Dec 9	Malaysia	1	$10^{5.2}$	0.054/0.078	<10	0812bTw	C/E/S African	V	FJ807893
13	2008 Dec 10	India	1	$10^{4.7}$	0.138/0.070	<10	0812cTw	C/E/S African	A	FJ807894
14	2009 Jan 31	Malaysia	2,15	$10^{4.9}$, ND	0.110/0.057, 2.896/0.599	<10, 640	0901aTw	C/E/S African	V	FJ807895

*qRT-PCR, quantitative reverse transcription–PCR; Ig, immunoglobulin; IFA, indirect immunofluorescent antibody; E1, envelope 1 (protein); ND, not determined; C/E/S, Central/East/South African.
†Date person returned to Taiwan or entered Taiwan.
‡Country that the person visited or native country of the person.
§Days postonset of illness that serum samples were obtained.
¶Values are patient sample optical densities at 405 nm.
